# Overview of Restrictive Cardiomyopathies

**DOI:** 10.14797/mdcvj.1078

**Published:** 2022-03-14

**Authors:** Smitha Narayana Gowda, Hyeon-Ju Ali, Imad Hussain

**Affiliations:** 1 Department of Cardiovascular Medicine, Houston Methodist Hospital, Houston, Texas, US

**Keywords:** restrictive cardiomyopathy, iron overload cardiomyopathy, idiopathic RCM, endomyocardial fibrosis, radiation-induced cardiomyopathy, Loeffler’s endocarditis, Fabry’s disease

## Abstract

Restrictive cardiomyopathy (RCM) includes a heterogeneous group of diseases that cause increased myocardial stiffness, leading to impaired ventricular relaxation and severe diastolic dysfunction. Given that it is the least common type of cardiomyopathy, it can be a diagnostic challenge due to its varied pathogenesis, clinical presentation, and diagnostic evaluation. In this review, we provide an overview of different etiologies of RCM and examine the diagnostic and treatment approaches for various types.

## Introduction

Cardiomyopathy is a disorder in which the heart muscle is structurally and functionally abnormal in the absence of coronary artery disease, hypertension, valvular heart disease, and congenital heart disease sufficient to explain the observed myocardial abnormality. Based on the morphological and functional phenotypes, cardiomyopathy can be subdivided into dilated cardiomyopathy (DCM), hypertrophic cardiomyopathy (HCM), restrictive cardiomyopathy (RCM), arrhythmogenic right ventricular cardiomyopathy, and unclassified cardiomyopathy (ie, Takotsubo and left ventricular noncompaction cardiomyopathies).^1^ While the exact prevalence of RCM is unknown, it is the least common type of cardiomyopathy encountered in clinical practice.^2^ Although there is no universally accepted definition of RCM, it has distinct hemodynamic and morphological characteristics that include severe diastolic dysfunction with restrictive filling patterns and elevated filling pressures from increased myocardial stiffness. Systolic function and left ventricular (LV) cavity size is normal or mildly reduced until advanced stages of the disease. RCM can involve left, right, or both ventricles with typically normal to slightly increased ventricular thickness. However, atrial enlargement is common due to increased atrial pressure.^3^

RCM has traditionally been subdivided into primary/idiopathic and secondary. Primary/idiopathic RCM can be familial or sporadic, and it is associated with a worse prognosis than secondary RCM. Secondary RCM stems from systemic disorders affecting the heart, such as amyloidosis, sarcoidosis, and iron overload. RCM can also be subdivided based on the histopathologic types, including infiltrative, non-infiltrative, storage disorders, and endomyocardial type (***Table 1***).^4^ To further add to the challenge of classifying cardiomyopathies, infiltrative diseases such as sarcoidosis and hemochromatosis can progress to DCM. Younger patients tend to have genetically inherited forms of RCM, such as storage disorders and hereditary hemochromatosis, whereas older patients tend to present with acquired disorders such as cardiac amyloidosis, secondary iron overload cardiomyopathy, and radiation-induced cardiotoxicity. In endemic areas such as Uganda, Nigeria, and India, endomyocardial fibrosis is more common.

**Table 1:** Causes of restrictive cardiomyopathy.


**Infiltrative** (Accumulation of substance between myocytes)	Amyloidosis (inherited/acquired) Hurler’s disease (inherited) Hunter’s disease (inherited) Gaucher’s disease (inherited) Sarcoidosis (acquired)

**Storage disorders** (Accumulation of substance within myocytes)	Fabry’s disease (inherited) Glycogen storage disease (inherited) Iron overload cardiomyopathy (inherited/acquired)

**Non-infiltrative**	Scleroderma (acquired) Idiopathic restrictive cardiomyopathy (acquired/inherited) Pseudoxanthoma elasticum (inherited)

**Endomyocardial**	Endomyocardial fibrosis (multifactorial) Hypereosinophilic syndrome (acquired) Endocardial fibroelastosis (inherited) Carcinoid syndrome (acquired) Drug induced: hydroxychloroquine, ergotamine, methysergide (acquired) Radiation-induced cardiomyopathy (acquired)


Since cardiac amyloidosis and sarcoidosis are discussed in detail throughout this issue, this review will focus on iron overload cardiomyopathy, Fabry’s disease, endomyocardial fibrosis, hypereosinophilic syndromes, and drug- and radiation-induced cardiotoxicity in greater detail.

## Clinical Manifestation and Physical Examination

Patients with RCM present with signs and symptoms of heart failure (HF), including dyspnea, fatigue, orthopnea, and paroxysmal nocturnal dyspnea that can worsen over months. Exercise intolerance is an early symptom because increased heart rate can lead to significant decline in diastolic ventricular filling and cardiac output. With right ventricular (RV) involvement, lower extremity edema and right upper quadrant pain can be present. Patients can also present with chest pain, syncope, and palpitations. Atrial fibrillation is common in RCM given the atrial enlargement, and thromboembolic complications are common. Examination findings include signs of left-sided HF such as pulmonary edema, third heart sound (S3), and right-sided HF signs such as elevated jugular venous pressure, pedal edema, ascites, and hepatomegaly. On examination of the jugular venous pulsations, prominent x and y descent can be a sign of RCM. With advanced disease, patients can have malnutrition, cardiac cachexia, and hepatic and renal dysfunction. Patients also present with manifestations of their underlying systemic disease process, which can serve as clues in diagnosis.

## Diagnostic Evaluation

RCM should be suspected in patients who have HF of unknown etiology and features of RCM on echocardiography. Physicians should assess for a history of amyloidosis, sarcoidosis, hemochromatosis, storage disorders, prior mediastinal radiation, use of hydroxychloroquine, family history of RCM, and HCM. In patients with signs and symptoms of RCM and increased LV thickness, it is important to assess for long-standing hypertension and HCM. Electrocardiogram can show p-wave abnormalities suggestive of atrial enlargement and nonspecific ST-T wave changes. Atrial fibrillation is the most common rhythm abnormality encountered in these patients. Other nonspecific findings include pseudo-infarct pattern, atrioventricular (AV) block, interventricular conduction delay, and premature atrial complexes. Low voltage or LV hypertrophy can be seen. ***Table 2*** provides an overview of the physical exam, laboratory, and imaging findings that point to RCM.

**Table 2:** Diagnostic features of primary and secondary RCM. LV: left ventricular; IOC: iron overload cardiomyopathy; HFE: hereditary hemochromatosis; LGE: late gadolinium enhancement; alpha-Gal A: alpha-galactosidase A; GI: gastrointestinal; AV: atrioventricular; RCM: restricted cardiomyopathy


TYPE	CLINICAL FINDINGS	LABORATORY INVESTIGATION/GENETIC TESTING	ECHOCARDIOGRAPHY	CARDIAC MAGNETIC RESONANCE IMAGING	ENDOMYOCARDIAL BIOPSY	TREATMENT

Idiopathic RCM	Any age group, family hx +/-, skeletal myopathy +/-	Genetic testing: mutations in sarcomere encoding proteins/desmin	+/- increased LV wall thickness		Myocyte hypertrophy, disarray, and interstitial fibrosis	Symptomatic treatment; advanced heart failure therapies

Iron overload cardiomyopathy	Primary: skin pigmentation, diabetes, liver dysfunction Secondary IOC: underlying hematological disorder	Elevated serum ferritin and transferrin saturation level Primary IOC: HFE gene mutation	+/- increased LV wall thickness Dilated cardiomyopathy at later stages	Decreased T2* relaxion time	Prussian blue staining+	Phlebotomy, iron chelators

Fabry’s disease	Cardiac manifestation: 30s, later in females	Absent or reduced leukocyte alpha-Gal A activity Genetic testing: gene encoding alpha-Gal A	Increased LV wall thickness	Midmyocardial LGE pattern in basal inferolateral wall	Concentric lamellar bodies in the sarcoplasm of myocytes	L-algalsidase beta

Endomyocardial fibrosis	Tropical countries, endemic disease, malnutrition Bimodal peak at 10 and 30 years	Eosinophilia +/-	Mural thrombus in apical and valvular pockets Obliteration and retraction of ventricles, endomyocardial thickening, AV valve regurgitation	Subendocardial diffuse LGE pattern from apex to valvular region, thrombus +/-	Fibrosis, +/- eosinophilic infiltration	Steroids in early phase, endomyocardial resection with valve repair or replacement

Hypereosinophilic syndromes	Skin rash, pulmonary, GI, neurological manifestations > temperate zones	Eosinophilia	Endomyocardial thickening, AV valve regurgitation, mural thrombus	Subendocardial patchy or diffuse LGE pattern, high intensity on T2-WI, +/- thrombus	Eosinophilic infiltrates, fibrosis	Steroids +/- Hydroxyurea/interferon alfa Imatinib: F1P1L1-PDGFRA mutation Endomyo-cardectomy with valve repair or replacement

Drug-induced RCM	Hydroxychloroquine		Increased wall thickness		Curvilinear bodies, lysosomes, myeloid bodies, glycogen granules and myocyte vacuolation seen on electron microscopy	Withdrawal of the drug

Radiation-induced RCM	Mediastinal radiation, latent period 10-15 years		Valvular calcification	Transmural or subendocardial LGE, perfusion defects +	Fibrosis	Symptomatic treatment


### Echocardiography

**Figure 1:**
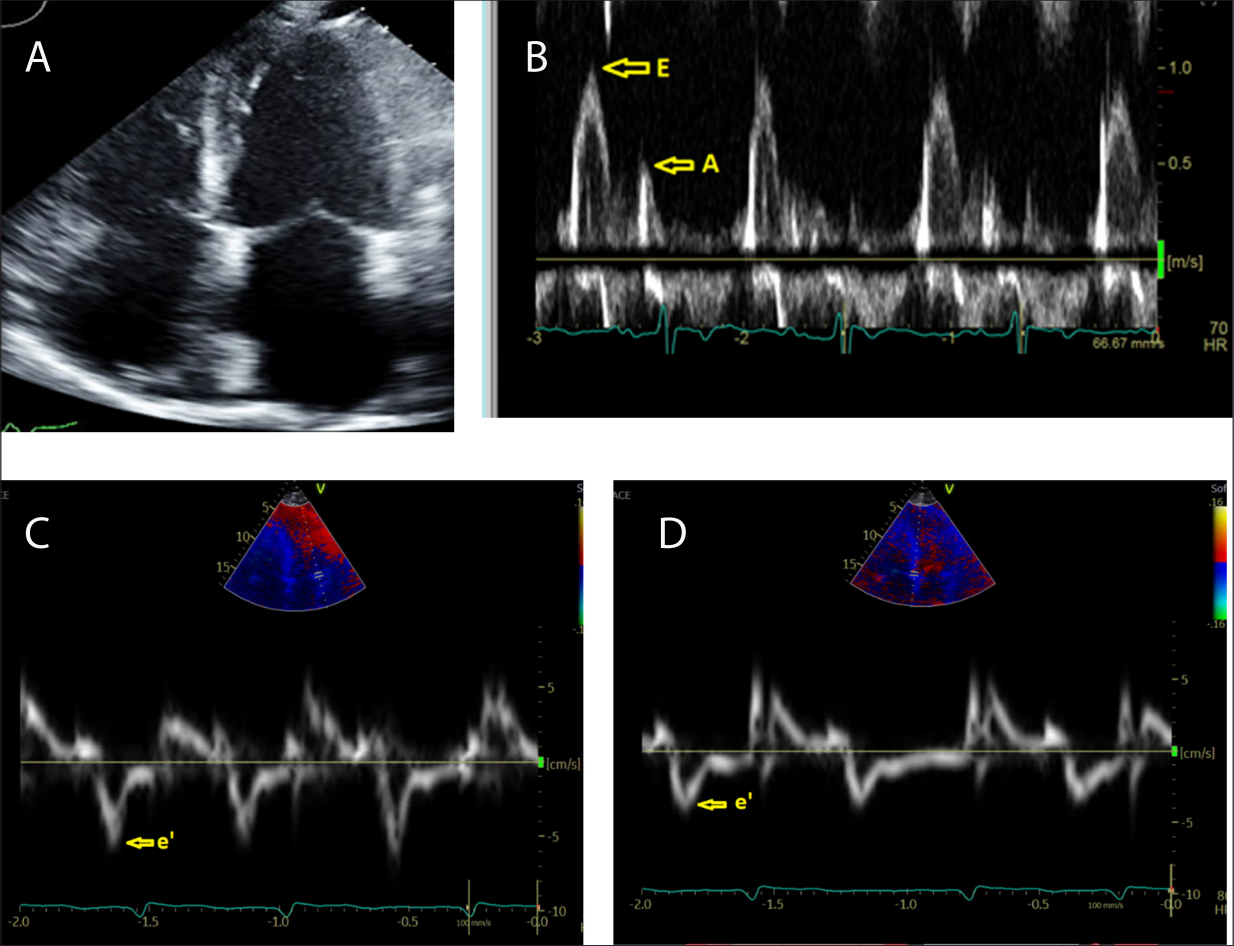
(**A**) Apical 4-chamber view showing biatrial enlargement. (**B**) Mitral inflow doppler showing increased E/A ratio (feature of diastolic dysfunction). (**C, D**) Lateral and septal mitral annular tissue doppler showing decreased e’ velocity.

Echocardiography is an essential tool in the initial evaluation of patients with RCM. Findings characteristic of RCM include normal or near-normal systolic function and cavity size, biatrial enlargement, and abnormal diastolic function. LV wall thickness is typically normal in non-infiltrative RCM but can be increased in patients with infiltrative and storage diseases. Abnormal diastolic dysfunction is noted on Doppler evaluation (***Figure 1***). It is important to note that diastolic dysfunction alone cannot be used to diagnose RCM, as this is commonly present in other forms of HF, including HF with reduced and preserved ejection (***Table 3***).^5^

**Table 3:** Echocardiography findings in restrictive cardiomyopathy. EF: ejection fraction; LV: left ventricular.


Normal or mildly reduced EF

Normal or slightly increased LV wall thickness

Normal or slightly decreased LV cavity

Biatrial enlargement

Diastolic dysfunction Increased E/A ratio Short E wave deceleration time Decreased mitral annuluse’ velocity Increased E/e’ ratio Hepatic vein flow reversalwith inspiration


### Invasive Cardiac Catheterization

In RCM, invasive hemodynamics often reveal elevated right and LV end-diastolic pressures (EDP), with LVEDP at least 5 mm Hg higher than RVEDP. Right atrial pressure is elevated with a prominent x and y descent, which represent rapid filling in the setting of high-pressure gradient between the right atrium and ventricle. Right ventricular systolic pressure (RVSP) can be > 50 mm Hg, and RVEDP is typically less than one-third of RVSP, resulting in a ratio of RVEDP/RVSP < 0.3. In contrast, with constrictive pericarditis, there is equalization of right and left ventricular EDP, and RVSP is typically < 50 mm Hg. The “dip and plateau” sign refers to the rapid decline in ventricular pressure during early diastole followed by a rapid rise and plateau of ventricular pressure as the stiff ventricle fills; this phenomenon is also present in patients with constrictive pericarditis. Whereas with constrictive pericarditis there is intrathoracic-intracardiac pressure dissociation with respiration, there is no inspiratory discordance in concomitant left and right ventricular pressures in RCM.^6^

### Cardiac Magnetic Resonance Imaging

Cardiac magnetic resonance imaging (CMR) is a noninvasive gold standard for assessing ventricular volumes, myocardial mass, and regional and global systolic function. CMR also plays a crucial role in the diagnosis of RCM etiologies given its enhanced tissue characterization of various patterns of late gadolinium enhancement (LGE). LGE and post-contrast T1 mapping sequences can be used to calculate extracellular volume, a quantitative marker of myocardial fibrosis. Conventional T2-weighted imaging and T2 mapping enable the detection of myocardial edema and inflammation. T2* relaxation time is used to assess iron overload cardiomyopathy. CMR can also help differentiate constrictive pericarditis from restrictive cardiomyopathy. In certain circumstances, CMR has decreased the need for endomyocardial biopsy.^7^

### Endomyocardial Biopsy

**When noninvasive studies have failed**, endomyocardial biopsy **(EMB) can be helpful in identifying the underlying etiology; furthermore**, the histopathologic findings can help identify specific infiltrative and storage disorders such as amyloidosis, hemochromatosis, Fabry’s disease, or idiopathic RCM, which is characterized by myocardial disarray and fibrosis. Risks associated with EMB are rare but include injury to the tricuspid valve apparatus leading to tricuspid regurgitation, RV free wall perforation malignant arrhythmias, or tamponade. Depending on where the biopsies are taken, the involved myocardium may not be sampled appropriately, leading to a false negative biopsy.^8^ In small studies, guidance with CMR or electroanatomic mapping has improved the sensitivity and specificity of EMB in diagnosing various types of cardiomyopathies and is an important subject for future large-scale trials.^9^

## Treatment

While there is no standard guideline-based medical therapy specific to RCM, treatment is targeted to the underlying disease process. Symptomatic treatment includes diuretics for relieving systemic and pulmonary congestion. Hypovolemia is poorly tolerated in RCM given a decrease in ventricular filling and decreased cardiac output. Although new therapies such as spironolactone, empagliflozin, and sacubitril-valsartan have shown some benefits in HF with preserved ejection fraction (HFpEF), these therapies have not yet been specifically studied in RCM patients. Supraventricular arrhythmias like atrial fibrillation are common and are poorly tolerated; rhythm control over rate control is preferred when feasible. Conduction abnormalities can be seen in infiltrative disease and may indicate pacemaker implantation. Patients with advanced HF should be referred for transplant evaluation early given the rapid progression of disease. Specific treatments for the individual disease are discussed below.

### Primary/Idiopathic RCM

The primary form of RCM is noninfiltrative disease, a rare disease that can occur at any age and can be familial or sporadic. Increased sensitivity of muscle filament to calcium and accumulation of desmin and collagen type III is thought to be the pathogenesis of primary RCM. The familial form of idiopathic RCM is usually autosomal dominant with incomplete penetrance. Various mutations in genes encoding troponin I and T, actin, myosin, and desmin have been associated with idiopathic RCM. RCM with hypertrophied LV walls demonstrates microscopic characteristics similar to that of HCM, and these two conditions are thought to be a different phenotypic expression of the same genotype.^10^ EMB of patients with idiopathic RCM can show nonspecific degenerative changes such as myocyte hypertrophy, disarray, and interstitial fibrosis, and the extent of the interstitial fibrosis can be mild to severe. The prognosis in children with idiopathic RCM is poor, with a median survival of 1 to 1.4 years, although heart transplant has improved overall survival.^11^ In a retrospective study of 94 patients with idiopathic RCM with a mean age of 64 years, 5-year survival was 64%; men aged > 70 years, left atrial enlargement > 60 mm, and higher NYHA class were each associated with poor survival.^12^ The overall prognosis in idiopathic RCM is poor, and early referral to a specialty center for advanced HF therapy is important.

### Secondary RCM

#### Iron Overload Cardiomyopathy

Iron overload cardiomyopathy (IOC) can be defined as excess iron deposition in the cardiac tissue, leading to early restrictive cardiomyopathy with diastolic dysfunction and later DCM. Systolic dysfunction can occur as the disease progresses. ***Table 4*** provides an overview of the diverse forms as well as diagnosis and management.

**Table 4:** Iron overload cardiomyopathy (IOC).


Etiology	Primary IOC	Hereditary hemochromatosis

	Secondary IOC	Hereditary anemia Sickle cell disease Thalassemia Sideroblastic anemia Acquired anemias Aplastic anemia Leukemias Myelofibrosis Myelodysplastic syndromes Stem cell transplantation End-stage renal disease Chronic liver disease Increased dietary intake

Diagnostic evaluation	Biomarkers	Serum ferritin > 200 ng/mL in premenopausal women or > 300 ng/mL in men and postmenopausal women Serum transferrin > 45% in men and > 55% in women

	Echocardiography	Early stage: diastolic dysfunction with pseudonormalization or restrictive filling pattern with or without atrial enlargement. Later stage: left and right cardiac chamber dilation with reduced left ventricular ejection fraction

	Cardiac magnetic resonance	T2* valve < 20 ms indicates IOC, < 10 ms indicates severe iron overload and poor outcomes


##### Etiology

IOC occurs mainly in two different scenarios: patients with hereditary hemochromatosis (HH) who have increased iron absorption, and patients receiving high parenteral iron (end-stage renal disease) or repeated blood transfusions for acquired or inherited anemias (eg, aplastic anemia, thalassemia, or sickle cell disease).^13^

Primary or HH IOC is an autosomal recessive disorder most commonly caused by HH gene mutation that regulates iron absorption. In HH, there is increased intestinal iron absorption and toxic accumulation in the body, leading to organ dysfunction. While it is the most common genetic disorder in White patients, there is low penetrance and disease manifestation, especially in women.^14^

**Figure 2:**
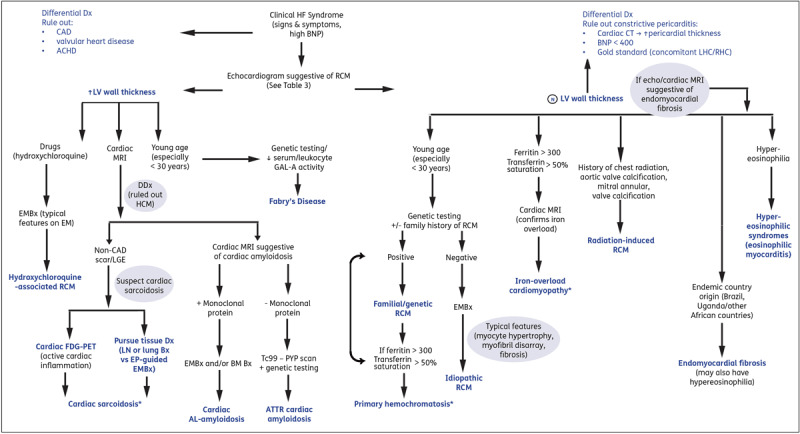
Suggested algorithm for evaluation of RCM. Please note that as RCM is a heterogenous disease process, there may be variability in clinical presentation (eg, cardiac hemochromatosis or sarcoidosis may also present with dilated phenotype and normal wall thickness).* This suggested approach is intended to assist with comprehension. Blue font indicates final diagnosis. Dx: diagnosis; DDx: differential diagnosis; CAD: coronary artery disease; HF: heart failure; ACHD: adult congenital heart disease; BM: bone marrow; EMBx: endomyocardial biopsy; RCM: restricted cardiomyopathy; MRI: magnetic resonance imaging; FDG-PET: fluorodeoxyglucose positron emission tomography; LV: left ventricular; Bx: biopsy; AL: light chain amyloidosis; ATTR: transthyretin amyloidosis; LGE: late gadolinium enhancement; Tc99-PYP scan: technetium-99 m pyrophosphate scintigraphy; AV: atrioventricular; GAL-A: alpha-galactosidase A; EP: electrophysiologic; LHC/RHC: left/right heart catheterization

Secondary IOC is more common, and one reason for the increased prevalence is improved survival from better treatment strategies in patients with hereditary anemias and other hematological disorders, but with an increased need for chronic blood transfusions.^15^ Cardiovascular disease is a major cause of death in these patients, so early recognition and treatment is important (***Figure 2***).^16^

##### Pathogenesis

When there is excess iron in circulation, transferrin becomes saturated and non–transferrin-bound toxic iron accumulates in hepatocytes, myocytes, endocrine glands, and other organs. In cardiac tissue, accumulation begins in the epicardium, spreads to the myocardium, and eventually involves the endocardium. This helps explain the preservation of systolic function until very late in the disease course.^17^ It can also affect the conduction system and lead to AV blocks as well as atrial and ventricular arrhythmias.^18^ In early stages of IOC, iron accumulation in the myocardium leads to diastolic LV dysfunction from the restrictive pathophysiology, which, if left untreated, leads to LV remodeling and progression to end-stage DCM. In some phenotypes, LV dysfunction leads to pulmonary hypertension, RV dilation, and right HF even in patients with preserved LV ejection fraction (LVEF).^19^

##### Clinical Features

Symptoms vary, but patients are usually asymptomatic in the early stages. Exertional dyspnea is the initial presenting symptom from restrictive pathophysiology, and as the disease progresses, patients can present with pulmonary edema, leg swelling, arrhythmias, and conduction abnormalities. A high degree of suspicion and clinical vigilance is required, especially in patients at increased risk, to identify cardiac involvement early in the disease process and prevent irreversible HF.^13^

##### Diagnostic Evaluation

Diagnosis can be challenging in the early stages of IOC. A thorough history, physical examination, electrocardiogram, and chest x-ray should be obtained in all patients with suspected disease, with regular cardiac follow-up and screening. Biomarkers, serum ferritin, and transferrin saturation are the initial clues to identify iron overload. If serum ferritin is > 200 ng/mL in premenopausal women or 300 ng/mL in men and postmenopausal women, or if transferrin saturation is > 45% in men and 55% in women, iron overload should be suspected.^20^ Serum ferritin can be elevated in other active inflammation conditions, and although these tests are important screening tools, they are not diagnostic of iron overload.

Echocardiography is an important imaging tool used to screen and follow patients with suspected IOC. The early finding includes diastolic dysfunction with pseudonormalization or restrictive filling pattern with or without atrial enlargement. In later stages, left and right cardiac chamber dilation with reduced LVEF is seen, and in some cases RV dilation and pulmonary hypertension, even with preserved ejection fraction; LV hypertrophy is generally not seen.^21^ While echocardiography is a great tool to screen and follow patients, it cannot detect cardiac iron content. CMR-derived T2* relaxation time has emerged as the main noninvasive imaging tool for quantitative assessment of cardiac iron content. T2* valve < 20 ms indicates IOC, and < 10 ms indicates severe iron overload and poor outcomes.^22^ Endomyocardial biopsy is no longer required routinely to assess patients with IOC; instead, CMR can be used to diagnose and monitor response to treatment.

##### Treatment

Early diagnosis is critical to prevent and treat iron overload and subsequent myocardial fibrosis. Dietary intervention, phlebotomy, iron chelating agents, and guideline-directed medical therapy for HF are the standard of care. Phlebotomy is used to decrease the iron load in patients with HH, beginning initially one or two times a week until the target ferritin level is achieved, followed by maintenance phlebotomies to keep ferritin at goal.

In patients with hypotension, malignancy, or anemia, iron chelating agents are used, including deferiprone, deferasirox, and parenteral deferoxamine. Use of iron chelating agents has been shown to prevent the development of IOC and has also been shown to reduce myocardial iron overload, improve cardiac function, reverse arrhythmias, and improve survival in patients with IOC.^23^ A heart transplant with or without liver transplant can be considered in patients with advanced HF, although it has been performed in a limited number of selected patients.^24^

#### Storage Disorders

Anderson-Fabry’s disease is an X-linked recessive lysosomal storage disorder with a mutation in the *GLA* gene that encodes alpha-galactosidase A (alpha-Gal A). There is a disruption in glycosphingolipid metabolism due to alpha-Gal A deficiency that results in intralysosomal accumulation of glycosphingolipids in various organs such as the heart, kidney, and nerves. Men are more commonly affected than women, and disease can present during childhood or early adulthood. Cardiac manifestations present later in life, the third decade in men, and can be silent until much later in women. In the classical variant, cardiac manifestations include arrhythmias, valvular abnormalities, and cardiomyopathy; however, the isolated cardiac variant (residual alpha-Gal A activity) presents later in life as LV hypertrophy. Echocardiography shows LV wall thickening, and tissue Doppler signals demonstrate reduced systolic and diastolic velocities of the mitral and tricuspid annulus. CMR may show a midmyocardial pattern of late enhancement of the basal inferolateral wall, sparing of subendocardium, and prolonged T2 relaxation time. For diagnosis in men, leukocyte alpha-Gal A activity is measured; however, this can be normal in heterozygous females, so genetic testing is recommended in women. EMB may be necessary in patients with uncertain genetic testing that demonstrates concentric lamellar bodies in the sarcoplasm of myocytes. Other than the standard medical therapy for HF, enzyme replacement therapy with agalsidase beta is the mainstay of treatment.^25^

#### Endomyocardial Fibrosis

Endomyocardial fibrosis (EMF) is an idiopathic disease that causes inflammation and fibrosis of the endomyocardium in one or both ventricles, leading to restrictive ventricular filling. EMF has geographic clustering and is prevalent in tropical countries such as Uganda, Nigeria, and Brazil. EMF has a strong association with poverty and malnutrition and is a disease of the young, with bimodal peaks at ages 10 and 30 years.^26^ The etiology of EMF has been debated, and various factors have been suggested, including parasitic infection, malnutrition, protein-poor diet, magnesium deficiency, high vitamin D, serotonin, cassava plant toxicity (contains linamarin, which liberates cyanide), eosinophilia, and immunological and genetic influence.^27^ EMF starts as an acute inflammation of the heart, leading to myocardial edema, eosinophilic infiltration, vasculitis, and fibrosis, collectively known as the active phase. Thromboembolic events are frequent during this phase, with mural thrombi occurring in apical and valvular pockets. Some patients go into fulminant cardiogenic shock, while others have recurrent inflammation that leads to fibrosis and, eventually, the chronic phase.^28^ In the chronic phase, there is fibrotic obliteration of one or both ventricles, and restrictive physiology, papillary muscle, and chordae tendineae involvement can cause valvular regurgitation. Biventricular involvement is seen in 50% of patients, followed by RV cardiomyopathy, and isolated left-sided disease is rarely seen. Patients present with dyspnea, paroxysmal nocturnal dyspnea, and lower-extremity swelling; when there is predominant right-sided involvement, marked ascites, elevated jugular venous distension, facial edema are common.^29^ Atrial fibrillation occurs in 30% of cases and predicts poor outcomes, especially in end-stage disease.^30^

Echocardiography is useful in diagnosis. Other than the typical restrictive filling pattern noted on Doppler assessment, characteristic findings include retracted ventricles with obliteration of the apex, endomyocardial thickening, atrial enlargement, and AV valve regurgitation. CMR can be useful in early disease to identify patients with active inflammation who may benefit from steroids; in chronic disease, LGE can demonstrate the extent of fibrosis, which correlates with prognosis and need for surgery.^31^ Medical therapy includes symptomatic treatment with diuretics and vasodilators, anticoagulation in patients with atrial fibrillation, and mural thrombus. Finally, management of advanced disease with endomyocardectomy, valve repair or replacement has shown to offer long-term benefits.^27^

## Cardiac Involvement in Hypereosinophilic Syndromes

**Figure 3:**
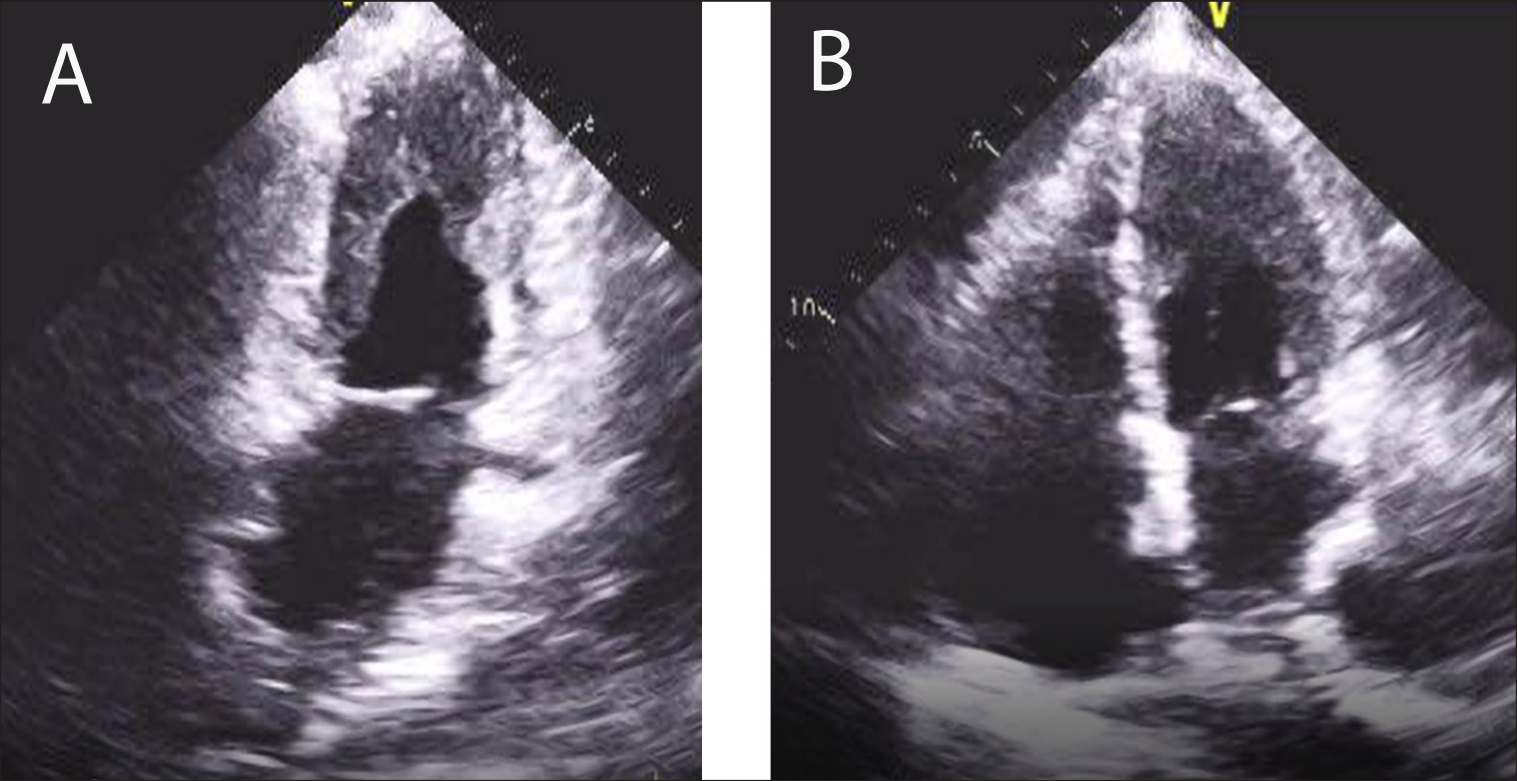
Apical 2-chamber and 4-chamber echocardiographic view showing biatrial enlargement, apical obliteration by thrombus.

Previously known as Loeffler’s endocarditis, hypereosinophilic syndromes (HES) are a group of disorders that cause persistent eosinophilia (absolute eosinophil count > 1.5 × 109/L) for longer than a month and cause eosinophil mediated end-organ damage. HES can be primary (stem cell, myeloproliferative disorders), secondary (parasitic and fungal infections, allergic conditions, solid tumors), or idiopathic (***Table 5***).^32^ Eosinophil-mediated endomyocardial damage can be seen on EMB, although the degree of cardiac injury does not correlate with the degree of eosinophilia. Cardiac involvement in HES evolves in three stages: an acute necrotic stage, an intermediate stage with thrombus formation, and a late fibrotic stage characterized by restrictive cardiomyopathy and AV valvular regurgitation depending on involvement of the chordae tendinae.^33^ In the acute phase, there is myocardial eosinophilic infiltration and degranulation that leads to myocardial necrosis and microabscess formation. At this stage, patients are generally asymptomatic except for those with fulminant eosinophilic myocarditis. Serum troponin elevation can be noted, and echocardiography is generally unremarkable.^34^ In the second stage, ventricular wall vascular damage leads to von Willebrand factor, tissue factor activation, and thrombus formation along with areas of damaged endocardium.^35^ Mural thrombus can be present in both ventricles, the ventricular outflow tract, and subvalvular areas. Distal embolization of the thrombus can lead to stroke, limb ischemia, and other thromboembolic consequences. In the third stage, persistent inflammation and necrosis will lead to subendocardial fibrosis and restrictive cardiomyopathy. Involvement of the valvular structures can lead to valvular incompetence from entrapment, rupture of the chordae tendinea, or fusion of the valves to the surface of the endocardium.^36^ Echocardiography in HES shows endomyocardial thickening/fibrosis, apical thrombus, biatrial enlargement, restrictive filling, and AV valvular regurgitation. CMR can differentiate different stages of the disease and can delineate intracardiac thrombus and the extent of fibrosis (***Figure 3***).^37^

**Table 5:** Hypereosinophilic syndromes. vWF: von Willebrand factor; AV: atrioventricular


TYPE OF SYNDROME	DESCRIPTION

Hypereosinophilic syndromes	Absolute eosinophil count > 1.5 × 10^9^/L for longer than 1 month and eosinophil-mediated end organ damage

Primary Secondary Idiopathic	Stem cell and myeloproliferative disorders Parasitic, fungal infections, allergic conditions, solid tumors

Cardiac involvement	Acute necrotic phase: eosinophilic infiltration, degranulation, inflammation and necrosis Thrombotic phase: activation of vWF and tissue factor on damaged endocardium and thrombus formation Late fibrotic phase: recurrent inflammation leading to fibrosis

Echocardiography	Acute stage: no changes unless fulminant myocarditis Thrombotic/fibrotic stage: endomyocardial thickening/fibrosis, restrictive filling pattern, AV valve regurgitation due to fibrotic involvement of the valve apparatus

Treatment	Treat the underlying condition Acute phase: Corticosteroids +/- hydroxyurea/interferon alfa Latent stage: heart failure management Endomyocardectomy, valve repair or replacement


EMB is the gold standard in the diagnosis of eosinophilic cardiomyopathy but is reserved for patients with an unclear diagnosis. Management strategies depend on the underlying condition; in the early stages of disease, high-dose corticosteroids can prevent disease progression to endomyocardial fibrosis. Steroid-sparing or second-line agents such as hydroxyurea, interferon alfa, methotrexate, and IL-5 blocker mepolizumab are other treatment options.^38^ Patients with Fip1-like1-platelet-derived growth factor receptor alpha mutation should be treated with tyrosine kinase inhibitor (eg, imatinib). Anticoagulation is not used prophylactically but is indicated in patients with thromboembolic events. Its use in patients with ventricular thrombus is debated because thrombus formation occurs at the damaged endocardium, and systemic anticoagulation may not suppress thrombosis. Vitamin K antagonists are preferred although there is no consensus on INR goal.^32^ In the fibrotic stage, some patients may need endomyocardectomy, valve repair, or replacement if there is valvular involvement. Bioprosthetic valves are preferred over mechanical valves due to the risk of thrombosis, even in patients on anticoagulation. Optimal medical management of the underlying disease is paramount as persistent eosinophilia is associated with poor outcomes and rapid valve deterioration. Endomyocardectomy in advanced restrictive cardiomyopathy has been shown to improve 5-year survival compared to medical management alone.^39^

## Drug-Induced RCM

Long-term use of chloroquine and hydroxychloroquine has been associated with cardiotoxicity. Risk factors are thought to be old age, female sex, long duration of hydroxychloroquine use (> 10 years), high dosage of the medication, renal dysfunction, and preexisting cardiac disease.^40^ These medications can cause RCM with or without conduction abnormalities. On echocardiography, there is increased ventricular wall thickness and restrictive filling pattern. Endomyocardial biopsy, which must include samples sent for electron microscopy, shows loss of architecture, curvilinear bodies, lysosomes, myeloid bodies, glycogen granules, and myocyte vacuolation. Drug withdrawal in some cases has shown complete resolution of EMB changes.^4^

### Radiation-Induced Cardiotoxicity

Mediastinal radiation has been associated with long-term cardiovascular sequelae, and these patients have an increased risk of coronary artery disease, valvular heart disease, congestive HF, arrhythmias, and pericardial disease. Radiation-induced cardiotoxicity typically occurs with a latent period of 10 to 15 years. Patients treated before 40 years of age are at increased risk given their longer period of survival.^41^ Radiation causes microvascular endothelial injury and inflammation that eventually leads to myocardial fibrosis, restrictive hemodynamics, and diastolic dysfunction. Systolic dysfunction may also be seen with concomitant use of anthracycline chemotherapy. Patients can be asymptomatic or can have exercise intolerance and symptomatic HF. Echocardiography demonstrates diastolic dysfunction, valvular calcification, and normal or reduced LVEF. Constrictive pericarditis physiology is present in some patients and may overlap with RCM, presenting a diagnostic challenge.^42^ Prevention by limiting cardiac radiation dose is important, and more novel techniques for treatment planning using rotational and multiple radiation sources, respiratory gating, or deep inspiration breath-hold techniques are now recommended to decrease cardiac radiation exposure.^43^ The treatment strategy for HF is symptomatic management. Heart transplantation can be considered in patients with advanced HF, although the United Network for Organ Sharing database study demonstrated that 5-year survival was lower than in transplants for other types of RCM, mainly due to higher postoperative mortality. Radiation-induced RCM with the end-stage disease was predominantly in younger females with previous cardiac surgeries.^44^

## Conclusion

Restrictive cardiomyopathy usually presents as HFpEF and has distinct echocardiographic and hemodynamic features. Given its varied etiology and clinical presentations, it can be a diagnostic challenge and requires a high degree of suspicion. Evaluation of patients with suspected RCM is guided by their history, presenting features, and clinical suspicion. Management involves treatment of the underlying condition in secondary RCM, symptomatic therapy for systemic and pulmonary congestion, and early initiation of advanced HF therapies when needed.

## Key Points

- Restrictive cardiomyopathy (RCM) is a spectrum of diseases that increase myocardial stiffness and lead to diastolic dysfunction.
- RCM can be primary/idiopathic or secondary to systemic disorders.
- Echocardiographic findings include biatrial enlargement, normal left ventricular cavity size, and systolic function with severe diastolic dysfunction.
- A high degree of clinical suspicion and disease-specific workup is required for diagnosis.
- Management includes symptomatic treatment and treatment of underlying systemic disease.
